# Supplementary material for: The relationship between maternal glucose concentrations, gestational diabetes mellitus, placental weight, and placental vascular malperfusion lesions: a retrospective study of a U.S. pregnancy cohort
Source: medRxiv. 2025 May 16:2025.05.14.25327646. Preprint. [Version 1] doi: 10.1101/2025.05.14.25327646 (PMC12132164; doi:10.1101/2025.05.14.25327646)
Supplement: 1 [file NIHPP2025.05.14.25327646V1-supplement-1.pdf]

medRxiv preprint doi: https://doi.org/10.1101/2025.05.14.25327646; this version posted May 16, 2025. The copyright holder for this preprint (which was not certified by peer review) is the author/funder, who has granted medRxiv a license to display the preprint in perpetuity.
It is made available under a CC-BY 4.0 International license .

Supporting information

S1 Figure. Placental pathology decision tree from Northwestern Memorial Hospital.
Abbreviations: APGAR= appearance, pulse, grimace, activity, and respiration; CMV=cytomegalovirus; DR=delivery room; HSV=herpes simplex virus; IUFD=intrauterine fetal demise; N=no; NICU=neonatal intensive care unit; PPROM=preterm premature rupture of membranes; SGA=small for gestational age; Y=yes

S1 Table. Maternal hypertension categories by ICD-10-CM.
All diagnoses were collapsed into the overall category "Maternal Hypertension"
Abbreviations: ICD-10-CM= International Classification of Diseases, 10th Revision, Clinical Modification; PE=preeclampsia; HTN=hypertension; HELLP: hemolysis, elevated liver enzymes, and low platelets; w/o=without

S2 Table. Gestational diabetes mellitus diagnosis by ICD-10-CM.
All diagnoses were collapsed into the overall category "GDM"
Abbreviations: ICD-10-CM= International Classification of Diseases, 10th Revision, Clinical Modification; GDM=Gestational diabetes mellitus; Gestatnl diab in chldbrth ctrl by oral hypoglycemic drugs=Gestational diabetes in childbirth controlled by oral hypoglycemic drugs

S3 Table. Associations between glucose challenge tests (per 10 mg/dL increase) and placental lesions (n=11,585).
† Poisson regression model adjusted for maternal age, race and ethnicity, parity, gestational age at delivery, and infant sex
Abbreviations: ARR=adjusted relative risk; CI=confidence interval; RR=relative risk; SE=standard error

S4 Table. GDM diagnostic criteria and total frequency.
† Diagnoses are not mutually exclusive (i.e. some women have >1 GDM diagnosis)
‡ Only from the current pregnancy, history of GDM diagnoses not included
Abbreviations: GDM=gestational diabetes mellitus

S5 Table. Associations between glucose groups and placental lesions (n=11,585).
The units for glucose challenge tests were 10 mg/dL
Interactions by infant sex and parity were not significant for any models (Wald test ≥ 0.1) and thus not included in this table
† Poisson regression model adjusted for maternal age, race (reference = NH White), parity (reference = 0), gestational age at delivery, and infant sex (reference = Female)
Abbreviations: ARR=adjusted relative risk; CI=confidence interval; GCT=glucose challenge test; GDM=gestational diabetes mellitus; RR=relative risk; SE=standard error

S6 Table. Associations between glucose challenge tests (per 10 mg/dL increase) and placental weight, a sensitivity analysis excluding patients diagnosed with maternal hypertension (n=10,832).
A total of 753 patients were diagnosed with maternal hypertension and were excluded
† Linear regression models were adjusted for maternal age, race and ethnicity, parity, gestational

medRxiv preprint doi: https://doi.org/10.1101/2025.05.14.25327646; this version posted May 16, 2025. The copyright holder for this preprint (which was not certified by peer review) is the author/funder, who has granted medRxiv a license to display the preprint in perpetuity.
It is made available under a CC-BY 4.0 International license .

age at delivery, and fetal sex
Abbreviations: AMD=adjusted mean difference; CI=confidence interval; MD=mean difference

S7 Table. Associations between glucose challenge tests (per 10 mg/dL increase) and categorical placental lesions (n=10,832).
A total of 753 patients were diagnosed with maternal hypertension and were excluded
† Poisson regression models were adjusted for maternal age, race and ethnicity, parity, gestational age at delivery, and fetal sex
Abbreviations: ARR=adjusted relative risk; CI=confidence interval; LGA=large for gestational age; RR=relative risk; SGA=small for gestational age

S8 Table. Associations between glucose groups and placental weight, a sensitivity analysis excluding patients diagnosed with maternal hypertension (n=10,832).
A total of 753 patients were diagnosed with maternal hypertension and were excluded
† Linear regression model was adjusted for maternal age, race and ethnicity, parity, gestational age at delivery, and fetal sex
Abbreviations: CI=confidence intervals; GDM=gestational diabetes mellitus; GCT=glucose challenge test; NH=non-Hispanic; SD=standard deviation

S9 Table. Associations between glucose groups and categorical outcomes, a sensitivity analysis excluding patients diagnosed with maternal hypertension (n=10,832).
† Poisson regression model was adjusted for maternal age, race and ethnicity, parity, gestational age at delivery, and fetal sex
Abbreviations: CI=confidence intervals; GDM=gestational diabetes mellitus; GCT=glucose challenge test
